# The prevalence and psychological relation of problem shopping: data from a large-scale sample from Turkey

**DOI:** 10.1186/s40359-021-00711-6

**Published:** 2022-01-03

**Authors:** Başak Ünübol, Barış Önen Ünsalver, Hüseyin Ünübol, Gökben Hızlı Sayar

**Affiliations:** 1 Department of Psychiatry, Erenköy Mental Health and Neurological Diseases Research and Training Hospital, University of Health Sciences, Istanbul, Turkey; 2 Department of Psychiatry, Medical Faculty, Üsküdar University, Istanbul, Turkey

**Keywords:** Problem shopping, Psychiatric distress, Affect, Attachment, IRT

## Abstract

**Background:**

The purpose of the present study was to comprehensively examine the measurement aspects, the prevalence, and the psychological correlates of problem shopping among a large-scale national sample of Turkish adults.

**Result:**

Participants (N = 24,380, 50% men, M age = 31.79 years, age range = 18–81 years) completed a questionnaire that comprised the Shopping Addiction Risk Questionnaire, the Brief Symptom Inventory, the Positive and Negative Affect Schedule, and the Experiences in Close Relationships-Revised. Results showed that 1.8% of the participants had probable shopping addiction. Being female, being younger, psychiatric distress, positive affect, negative affect, anxious attachment, and avoidant attachment were positive correlates of problem shopping.

**Conclusion:**

The results of this large sample size study suggest that shopping addiction is not a rare condition in Turkey. Further research is needed to understand different motives that underlie the problematic shopping behavior in the young and female population in comparison to older and male populations. Preventive programs or any interventions for people with PSB needs to address regulation difficulties and development of healthy strategies to cope with psychiatric distress.

**Supplementary Information:**

The online version contains supplementary material available at 10.1186/s40359-021-00711-6.

## Introduction

Shopping is part of everyday life but it may become problematic when it goes beyond meeting the shelter, nutrition, education, health, and recreational needs of the person and starts to limit the person's personal and social life and cause the person to be financially and morally negatively affected. In the globalized and hyperconnected world of the twenty-first century, excessive shopping has become a problem for all societies. What distinguishes shopping addiction (SA) phenomena from ordinary consumers, collectors and hoarders is that they focus mainly on the purchasing process and the emotions this process evokes, not specifically on the product purchased. For this reason, they often do not use the products they buy or discard them [1].

SA is not included as a distinct disorder in the current psychiatric diagnostic systems yet. Problematic shopping behavior (PSB) was defined as “oniomania '' by both Bleuler and Kraepelin in the early twentieth century [2]. However, it has been unclear whether this is a problem with impulse control, or a variant of obsessive–compulsive disorder or an addictive disorder. In accordance with that, various different names such as “oniomania,” “compulsive shopping,” “compulsive consumption,” “impulsive buying,” “compulsive buying,'' “compulsive spending” and “problematic shopping behavior” have been used to address the same clinical picture. In recent years, growing literature and clinical observations support the definition of PSB as a behavioral addiction like internet addiction, exercise addiction, and pathological gambling [3–6]. According to Lejoyeux and Weinstein, who define shopping addiction, purchasing behavior comes in an uncontrollable and repetitive form, the person spends most of his time shopping or imagining the shopping act; he always buys more than he had planned and continues his shopping behavior despite the negative consequences of shopping [7].

Griffith’s described components of addiction as salience, mood modification, tolerance, withdrawal symptoms, conflict and relapse [8]. PSB fits into this model. Therefore, “Problematic Shopping Behavior” will be used throughout the text to refer to any type of PSB. There is a constant preoccupation with shopping in PSB cases. Although they do not always purchase a new product, they devote a significant amount of time scrolling through online shopping sites or reading comments about certain products and they may neglect their work. In the early stages, subjects with PSB feel positive emotions when they shop [9]. Over time, shopping becomes a solution to get away from negative emotions [10]. Despite the feelings of guilt and regret after the shopping act, the person has difficulty controlling the shopping bouts as the problem progresses. As a result of the unstoppable shopping activity, family conflicts and relationship problems with the spouse arise, job performance may decrease, and increasing debts and related legal problems may even come down to illegal acts to overcome financial problems.

Quality of life may decrease and secondary psychiatric problems may occur. People with SA are generally aware of their problems [2]. Most are worried about their debt associated with shopping problems [11]. Nevertheless, they rarely seek help. They prefer to be alone while shopping because the presence of someone who doesn’t share the same positive feelings as themselves can be shame inducing.

Cross-sectional studies indicate that SA(shopping addiction) is chronic, although fluctuations are observed in its severity and intensity [11, 12]. In some cases of PSB, the problem is continuous and does not improve for more than a month, while in the other part, the disorder progresses periodically. Some cases encounter these periods every hour, while others occur once a month.

The negative consequences of PSB (eg, distress, impairment) and the underlying psychological and neuropsychological mechanisms have emerged in several studies [13–15].

### Measurement of problem shopping

Different scales for assessing problematic shopping behavior have been developed since the late 1980s. Some of these scales approach the problem either from the compulsive buying or impulse-control paradigm perspective [16–18] and do not assess components of behavioral addiction[8]. Although Bergen Shopping Scale [3] is among the widely used scales, it was not used in our study because it was developed with the data of the participants in Norway and there is no validity and reliability study for Turkish culture. When the cultural background, country and language of the new target population are different from the culture in which the scale was developed, the scale needs to be culturally adapted [19]. There is only an adaptation of the scale to Compulsive Online Buying Behavior in Turkey. This scale, on the other hand, is not sufficient to evaluate shopping addiction in general terms, to determine the prevalence of shopping addiction in Turkey and to define the nature of purchasing profiles in our study. Therefore, the need to use a new tool has emerged in our study.

### Prevalence of problem shopping

The inconsistency with the definition, naming and measurement of the problematic behavior has yielded different results in epidemiologic studies. The rate of the prevalence of SA in epidemiological studies was found to be between 1.1 and 20% [9, 20–23]. In a meta-analysis of 40 epidemiological studies on the shopping problem, the point prevalence was found to be 5% [24].

In recent years, CBSD has become important to public health. Prevalence estimates of around 5% in populations in different cultures indicate that this is a common condition [20, 21, 25]. Also the shopping process has changed with the advent of the internet. E-retail shopping has increased over the years and constituted 11.9% of all retails worldwide in 2018. In Turkey, where this study was conducted, the rate of online shopping was reported as 29.3% between the ages of 16–74 in 2018 [26]. As the amount of this shopping increases, the discomfort associated with online shopping also increases. Online problematic shopping behavior has also been shown to include known predictors of behavioral addictions in general: low self-esteem, low self-regulation, negative emotional state, and female gender [27]. It is possible that it is easier to lose control during online shopping than in-store shopping compared to offline shopping [28]. In our study, no questioning was made in terms of online and in-store shopping, and problematic shopping behavior was evaluated in general terms.

### Correlates of problem shopping

In clinical and field studies, it has been reported that 80–95% of the SA cases are women [20, 25]. It is stated that women, youth, and people with psychological disorders [29] are particularly susceptible to problematic shopping behaviors [30]. However, in some studies there was no effect of gender on SA [23, 31].

SA cases tend to be younger [20, 22, 23, 25, 32]. Lejoyeux et al. [33] found that fewer women (66%) with SA were married than the control group (85%). Most studies have failed to show any effects of sociodemographic variables like education, relationship status, income or employment on SA [20, 34].

SA is rarely seen as an isolated problem. In cases with shopping addiction, first and second axis disorders accompany the picture with a rate of 90% [23]. Mood disorders, anxiety disorders, eating disorders, hoarding disorder, impulse control disorder, gambling addiction, and substance use disorders are the most common psychiatric problems accompanying shopping addiction [35]. In the literature, it has been reported that a mood disorder accompanies SA in 21–100% [23, 36, 37]. In McElroy et al.’s [38] study, patients stated that when they were depressed, only shopping made them feel good. In a study from Brazil, Mattos et al. [39] reported that among the 171 patients with compulsive buying 164 had at least one psychiatric comorbidity, with anxiety and mood disorders being the most frequent. The decline in cognitive functions in depression and the need for rewarding behavior may facilitate shopping behaviour. “Retail therapy” stands out as a popular method for dealing with negative emotions in society.

People who have difficulty in regulating intense emotions whether negative or positive tend to act impulsively which puts them under risk for addictive disorders [40]. Shopping has a mood modifying effect as suggested in the multicomponent model of addiction [8]. It has been reported that people with SA might experience both positive and negative emotions before the purchase [41, 42]. Act of shopping or being occupied with shopping might have a temporary effective regulatory function.

Attachment style and shopping addiction relationships have been studied rarely. Main idea of attachment theory as proposed by Bowlby is that every human being has an innate need for psychological security that is provided by the care and protection of their attachment figure. People seek out proximity to their attachment figures, particularly when they are distressed. If a person is securely attached the attachment figure is perceived as loving, approving and a close person that can be trusted at any time. In contrast, the attachment figure is perceived as cold, distant and unreliable when a person has avoidant attachment. Therefore, they withdraw themselves when they are distressed in order not to be disappointed. Anxious attachment is characterized by a perception of the attachment figure as an inconsistent and confusing person who might be warm, loving and dependable at certain times and cold, distant and undependable at other times. Thus, they seek constant encouragement from their significant others. Attachment insecurity is related to increased psychiatric distress and difficulties in affective regulation [43]. Anxiously attached individuals might use inanimate objects as a means to feel socially connected and secure [44]. This is similar to what Winnicott called a “transitional object” such as a toy or a blanket which the child attaches to during the separation stage from the primary attachment figure. Transitional object attachment helps the child feel safe and secure when the primary caregivers are unavailable [45]. Negative affect states related to insecure attachment might trigger an increased attachment to and the need to possess inanimate objects. Shopping is also at times a social activity for individuals where they interact with sales persons. Therefore, especially those with anxious attachment might be expected to turn to shopping when they feel the need to connect while avoidantly attached individuals might refrain from shopping to avoid any social proximity.

### The present study

This survey study was conducted to examine a large-scale representative sample from different parts of Turkey utilizing a new instrument that was aimed to reflect the six components of behavioral addiction criteria to assess shopping addiction. More specifically, it psychometrically defines the employment of item response theory (IRT), a cut-off point for the population of interest, to accurately assess disordered buying prevalence. Furthermore, it employs latent class analysis (LCA) to define the optimum number of disordered shopping profiles, as well as the nature of their differences for that specific population. The study describes the intensity of the associations between shopping addiction and some psychological phenomenologies. Regarding the literature research on the correlates of shopping addiction, in the present study it was hypothesized that psychiatric distress, negative affect, and attachment styles would all be positively associated with shopping addiction.

## Methods

### Participants and procedure

Initially, 24,494 adults from the Turkish community filled out paper-and-pencil questionnaires. Inclusion criteria for participation was being over 18 years of age, and not having a mental illness that prevents the individual from completing the questionnaires. Participants' pre-diagnosis of mental illness was confirmed by clinical psychologists' interviews and participants' self-reports. Interviews were conducted with the participants to screen for other mental health problems that the clinic might present, such as manic episodes, similar to shopping addiction. Exclusion criteria- lack of education, presence of active mental disorder such as manic episode or psychosis, which may prevent completing the interview. The study was carried out in 79 different cities all over Turkey by 125 clinical psychologists via taking participants’ informed consent for participating in the study voluntarily and anonymously. Our study was introduced by clinical psychologists. Each clinical psychologist reached his environment from different sources and reached the participants face to face with the snowball sampling method. The participants were included in the study consecutively. The sample was planned based on the NUTS (nomenclature of territorial units for statistics) classification. NUTS is a hierarchical system for dividing up the economic territory of the European Union. The individuals residing in 26 NUTS3 regions of Turkey participated in the study. The final sample consisted of 24,380 participants (12,249 men and 12,131 women; *M*_*age*_ = 31.79 years, *SD*_*age*_ = 10.86; range = 18–81 years) who did not have unreliable responses and/or too much missing data. This article is part of a larger survey study on different behavioral addictions’ prevalence in Turkey.

### Measures

*Shopping Addiction Risk Questionnaire (SHARQ):* The unidimensional SHARQ (see Additional file 1: Appendix) was developed to assess shopping addiction (e.g., “*If I stop shopping, it can be triggered again and I can continue to pretend like I never left*.”) The scale consists of six items that assess components (Griffiths, 2005) of six addiction-like symptoms (salience, withdrawal, mood modification, conflict, tolerance, relapse) [8]. Items (0 = *never,* 10 = *always*) were averaged to create an index of shopping addiction (Cronbach’s α = 0.90). In the scoring of the SHARQ scale, the score of each item is calculated as 10, and 55 and above are classified as problematic shopping behaviour.

*Brief Symptom Inventory (BSI):* The Turkish form [46] of the 53-item BSI [47] assess five symptoms: anxiety (e.g., “*Suddenly scared for no reason.*”), depression (e.g., “*Feeling lonely.*”), negative self concept (e.g., “*Feelings of worthlessness.*”), somatization (e.g., “*Trouble getting your breath.*”), and hostility (e.g., “*Having urges to beat, injure, or harm someone.*”). Items (1 = *almost never*, 5 = *almost always)* were averaged to create a general index of global severity to assess general level of psychiatric distress (α = 0.95).

*Positive and Negative Affect Schedule (PANAS)*: The Turkish form [48] of the 20-item PANAS [49] was used to assess positive (e.g., “*enthusiastic*”, “*determined*”) and negative affect (e.g., “*alert*”, “*ashamed*”) at a given point in time. Items (1 = *very slightly*, 5 = *extremely*) were averaged to create indices of positive affect (α = 0.85) and negative affect (α = 0.83).

*Experiences in Close Relationships-Revised (ECR-R)*: The Turkish form [50] of 36-item ECR-R [51] was used to assess anxious (e.g., “*When my partner is out of sight, I worry that he or she might become interested in someone else.*”) and avoidant attachment (e.g., “*I am nervous when partners get too close to me.*”). Items (1 = *strongly disagree*, 7 = *strongly agree*) were averaged to create indices of anxious (α = 0.83) and avoidant attachment (α = 0.85).

### Statistical analysis

Exploratory factor analysis (EFA), confirmatory factor analysis (CFA), and Item Response Theory Graded Models (IRTGM) were used to examine the structure validity and cut-off score of the SHARQ. Root mean square residuals (RMSEA), standardized root mean square residuals (SRMR), comparative fit index (CFI), and goodness of fit index (GFI) were checked to determine goodness of fit in CFA. According to Hu and Bentler (1999), RMSEA and SRMR lower than 0.05 indicate good fit and RMSEA and SRMR lower than 0.08 suggest adequate fit; CFI and GFI higher than 0.95 is good and CFI and GFI higher than 0.90 is acceptable [52]. Frequency and descriptive statistics were used to calculate ratios, mean scores, and standard deviations of the study variables. Pearson correlation analysis was used to examine correlation coefficients among study variables. To examine predictors of shopping addiction, hierarchical regression analysis was applied. Statistical analyses were utilized running SPSS 23.0, AMOS 23.0, and Mplus 7.0 software.

## Results

### Scale development and prevalence of shopping addiction

Kaiser–Meyer–Olkin measure and Barlett’s test of sphericity (0.87; *p* < 0.001) in EFA suggested a one-factor solution. Principal component analysis indicated all items had high loads (communalities ranging between 0.57 and 0.75), explaining 67.29% of the total variance. EFA results were evaluated performing CFA. Goodness of fit indices (χ^2^ = 2422.50, *df* = 6, *p* < 0.001, RMSEA = 0.13 CI 90% [0.12, 0.13], SRMR = 0.03, CFI = 0.97, GFI = 0.97) stipulated mostly good fit to the data. According to the standardized regression weight (ranging between 0.65 and 0.86), all items had a significant role in the scale. Optimum number of categories in the SHARQ were determined via performing LCA. Successive models were compared considering fit and entropy values to evaluate the optimum number of profiles that best described a population. Analyses identified 4 classes for SHARQ. In order to calculate the cut-off score of the scale, IRTGM was used via transforming each calculated raw score into the scale equivalent of the latent factor assessed. Expected A Posteriori Measures under Rasch model conditions were used for these assessments. Eventually, any score that exceeded 2 standard deviations above the mean of the latent factor calculated were considered as threshold. Moreover, item difficulty and discrimination parameters across all items were calculated.

As a result of the analyses, it was found that those who scored 55 and higher on the SHARQ could be categorized as addicted to shopping. Accordingly, %1.8 of the participants were at very high risk for having a shopping addiction.

### Correlation and hierarchical regression analyses

Table 1 illustrates mean scores, standard deviations, and correlation coefficients of the study variables. Shopping addiction was positively correlated with psychiatric distress (r = 0.21, *p* < 0.001), positive affect (r = 0.08, *p* < 0.001), negative affect (r = 0.16, *p* < 0.001), avoidant attachment (r = 0.07, *p* < 0.001), and anxious attachment (r = 0.18, *p* < 0.001).

**Table 1:** Mean scores, standard deviations, and Pearson’s correlation coefficients of the study variables

		1	2	3	4	5	6
1. Problem shopping		–					
2. Psychiatric distress		.22*	–				
3. Positive affect		.08*	− .15*	–			
4. Negative affect		.16*	.58*	− .10*	–		
5. Avoidant attachment		.07*	.24*	− .28*	.23*	–	
6. Anxious attachment		.18*	.44*	− .10*	.37*	.21*	–
	*M*	16.00	98.20	30.42	19.46	60.27	60.21
	*SD*	14.43	29.04	7.97	6.83	19.23	18.36

^*^p < .001

Table 2 contains the results of hierarchical regression analysis. Gender and age comprised the Block 1. In Block 2, psychiatric distress, positive and negative affect, and adult attachment styles were included into the equation. Being women (β = 0.13, *p* < 0.001), being younger (β =  − 0.11, *p* < 0.001), psychiatric distress (β = 0.14, *p* < 0.001), positive affect (β = 0.13, *p* < 0.001), negative affect (β = 0.02, *p* < 0.01), avoidant attachment (β = 0.04, *p* < 0.01) and anxious attachment (β = 0.09, *p* < 0.001) were positively associated with shopping addiction. The regression model predicted 10% of the variance in shopping addiction (F_7,24220_ = 374.82, *p* < 0.001).

**Table 2:** Hierarchical regression analysis predicting problem shopping

Model	B	SE	β	*t*	ΔR^2^
Block 1 (R^2^_Adjusted_ = .04; ^F^_(2,24225)_ = 543.96; *p* < .001)					.04
Gender^a^	3.84	.18	.13	21.12**	
Age	− .20	.01	− .15	− 24.28**	
Block 2 (R^2^_Adjusted_ = .10; ^F^_(7,24220)_ = 374.82; *p* < .001)					.06
Gender^a^	3.71	.18	.13	20.90**	
Age	− .14	.01	− .11	− 17.13**	
Psychiatric distress	.07	.00	.14	17.93**	
Positive affect	.23	.01	.13	19.94**	
Negative affect	.05	.02	.02	3.16*	
Avoidant attachment	.47	.09	.04	5.31*	
Anxious attachment	1.29	.10	.09	13.12**	

B = Unstandardized regression coefficient; SE = Standard error; β = standardized regression coefficient; a) Men = 1, Women = 2; *p < .01, **p < .001

**Fig. 1:**
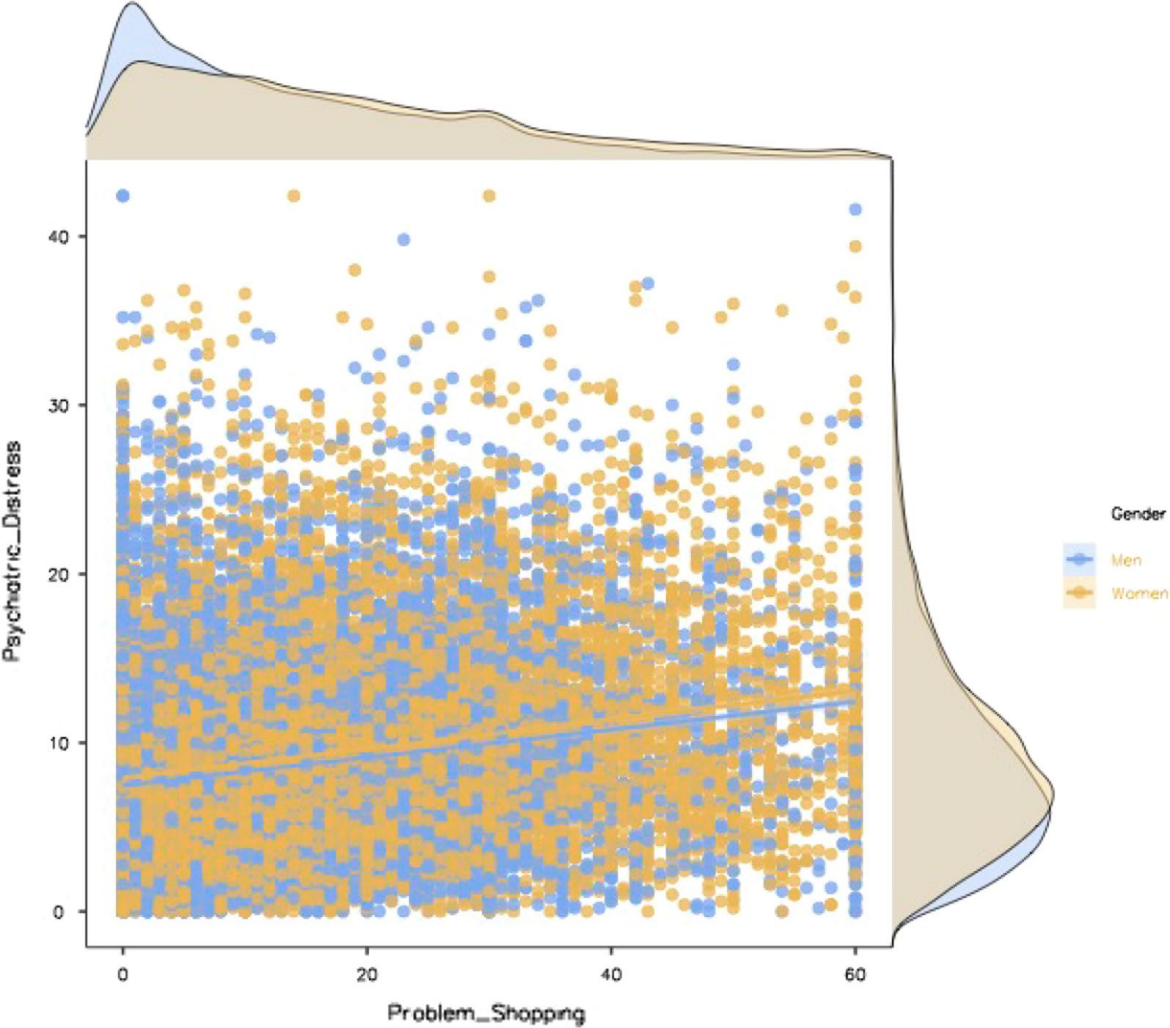
Scatterplot diagram of the correlation between psychiatric distress and problem shopping by gender

Figure 1 shows the Scatterplot diagram of the correlation between psychiatric distress and problem shopping by gender.

## Discussion

In the present study, we tested the psychometric properties of a newly developed short-scale for assessing shopping addiction, and examined the prevalence and psychological predictors of shopping addiction in relation to sociodemographic factors in a large-scale Turkish sample (N = 24,380). Being female, being younger, psychiatric distress, positive affect, negative affect, anxious attachment and avoidant attachment were positively associated with shopping addiction.

### Measurement of problem shopping

We preferred a new questionnaire **‘***Shopping Addiction Risk Questionnaire (SHARQ)’* for this study. Impulsivity is frequent among people with PSB which necessitates the use of a very brief scale. Therefore, a questionnaire with concise, clear and well targeted questions that was based on components of behavioral addiction and that could be answered by most of the subjects was fit for the scale of the current study.

### Prevalence of problem shopping

1.8% of the participants in our survey study were found to be at very high risk for having shopping addiction as assessed by SHARQ. The point-prevalence for SA in the meta-analysis study by Maraz et al. (2016) was 5% [25]. Though our finding is lower than this, it can still be said that PSB is not rare and an issue of concern in Turkey. Turkey has a population of more than 83 million people (Turkish Institution of Statistics, https://data.tuik.gov.tr/Bulten/Index?p=Istatistiklerle-Kadin-2020-37221) which makes it probable that at least 1.5 million people in Turkey might be suffering from SA.

### Predictors of problem shopping

In our study, being a woman was positively associated with shopping addiction. This is in accordance with most of the previous studies on problematic shopping disorders [22, 24]. There is a gender stereotype where shopping is regarded as a gender appropriate behavior for women which might make it easier for women to normalize their shopping behaviors. When the literature on gender and consumption is examined, it is claimed that women generally have more purchasing behavior and spend more time shopping [53, 54]. This situation, which is also referred to as the "feminization of shopping" in the literature, has been used to describe women's shopping experiences. The fact that the advertising industry specifically identifies women as the target audience shows that women participate in consumption more frequently [55]. Dittmar states that buying behavior is driven by materialistic values and discrepancies in identity, so she suggests that buying can be conceptualized as an identity seeking behavior [22]. Either through normalization or fulfillment of identity needs, women are under increased risk for the development of PSB in comparison to men.

Being younger was positively associated with shopping addiction. The younger the person the more he needs to be seen, accepted and appreciated especially by his peers and also by the rest of the community [56, 57]. Shopping might serve these needs well. What is more, the free trade globalised economy and the online hyperconnected state of the world that young people are born into makes it easier for them to explore and be easily tempted to shop for various products from any part of the globe. Accessibility of the product smooths the path to purchasing it. Young people may be more impulsive and have more difficulties in emotional regulation in comparison to older people which are both risk factors for behavioral addiction development [24, 40]. In many studies, it has been reported that SA starts at the end of adolescence and becomes a significant problem in the 30 s [7, 11, 13, 20]. This might suggest that the period in which the individual reaches economic independence and starts earning his own money is compatible with the development of tolerance to shopping behavior.

In our study psychiatric distress was positively related to PSB. Some people might turn to shopping to relieve their psychiatric distress [41]. As has been reported SA has a high comorbidity with other psychiatric disorders [20, 23]. In order to predict that PSB is an addiction rather than a symptom of another mental health condition and to avoid this possible confusion/limitation, individuals with additional psychiatric diagnoses such as manic episodes were not included in our study.

In a study by Müller et al. [42], 25 participants with compulsive buying (CB) were monitored with a handheld computer where they noted their affective states using the PANAS scale in relation to the CB episodes. It was reported that negative affect increased before a CB episode and decreased after the purchase, whereas positive affect decreased before CB episode, but did not increase after the CB episode. Bellini et al. [58] reported that among 316 mall customers, a higher positive effect before entering the shopping mall was related to more impulse buying. Three clusters of buyers were identified in a sample of 419 compulsive buyers depending on the affective states prior to buying episodes [59]. “Escape seekers” buy in response to negative emotions, “excitement seekers” buy to get stimulated when they feel bored and the act of buying in “low affect management buyers” is not affected by any type of emotions prominently. In our study, both negative and positive effects were positively correlated with PSB. Though we did not specifically ask for the affective state of the subjects prior to their shopping experiences, our findings can still support the relation between positive and negative affective states and the tendency for problematic shopping behavior.

Anxious attachment is associated with distress intolerance and poor impulse control, both of which are related to uncontrolled buying. Excessive buying in anxious attachment may be a maladaptive coping strategy for emotion dysregulation [60]. In contrast, avoidantly attached individuals dismiss their needs for social connection and they do not attach to objects for support [44, 60]. In Keefer et al.’s study (2012), they hypothesized that increased attachment anxiety would mediate the effect of the prime on object attachment, but attachment avoidance would not. The results of their main study revealed that attachment avoidance also predicted object attachment. However, the effect of priming on an object was mediated only by attachment anxiety, not attachment avoidance. In our study, both anxious and avoidant attachment were positively associated with PSB, but further mediation analysis was not performed like Keefer et al.’s study. We did not specifically ask about online shopping behaviour in our study which is a solitary and somewhat discrete way of shopping. So it could be said that online shopping might form a safe ground especially for individuals with avoidant attachment patterns.

Various factors have been examined in previous studies on behavioral addictions. It is stated that there is an inverse relationship between addiction and healthy attachment styles. Flores [61] interpreted addiction as an attachment disorder, a maladaptive and late transition in young adulthood. Studies examining the relationship between behavioral addictions and attachment support the relationship between social media addiction and two types of insecure attachment (ie, anxious and avoidant) [62]. In some studies investigating the relationship between attachment style and internet addiction, it is stated that participants with insecure or ambivalent attachment style are more prone to pathological internet use compared to participants with secure attachment [63, 64]. There are very few studies investigating the relationship between attachment style and shopping addiction [65]. The findings of our study related to attachment styles were remarkable in this context.

Attachment styles and object attachment have been studied mainly in patients with hoarding disorder but not in people with problematic shopping behavior [66] which makes the current study unique. Hoarding disorder is different from shopping addiction because even though the stuff might have been gathered as a result of shopping addiction the main issue is that of being unable to discard possessions. Furthermore, hoarded materials might not have been acquired as a result of shopping. Norberg et al. [60] compared objects with hoarding disorder and compulsive buying in terms of object attachment and anthropomorphic object choice, but not in terms of personal attachment styles. In their study, those objects who were primed to recall an instance with a significant other where they felt unsupported were more likely to acquire comfort items. This finding supports both the stress relieving role of shopping and also fulfillment of attachment needs of proximity by purchasing a comfort item. Avoidantly attached individuals would be expected to turn to comfort item shopping when they are distressed which might explain the association of avoidant attachment and PSB in our study. In future studies differences in shopping tendencies of anxious and avoidant people can be explored.

### Limitations and conclusion

The cross-sectional nature of the present study limits the interpretation of the findings despite the large sample size. Further longitudinal studies will help to understand changes in psychiatric symptoms relating to PSB over time. Self-report questionnaires were used in the study which might have led to recall and social desirability biases. A recent study suggested that online and in-store shopping addiction are not completely two different entities and they do overlap[28]. However, people with either type of PSB show somewhat different patterns of shopping that might interfere with the sociodemographic features of PSB. Online-shopping behavior was not questioned specifically in the study which might have affected some of the socio-demographic findings.

This study was done before the COVID-19 pandemic. Panic bulk buying[67] and compulsive buying behaviour has increased during the pandemic[68]. New studies can shed light on whether this compulsory increase in e-commerce or panic buying has changed the prevalence of shopping addiction. Even though there are some limitations, this is the first study that empirically explores the psychological predictors of shopping addiction with the largest sample until now from different areas of Turkey.

## Conclusion

Our study provides important contributions to the literature in this field by evaluating the variables associated with problematic shopping behavior as well as evaluating attachment styles. The SHARQ scale we used in our study consists of six items that evaluate the components (Griffiths, 2005) of addiction-like symptoms (satelliteness, withdrawal, mood change, conflict, tolerance, relapse). It was observed that this scale predicted problematic shopping behavior. The results of this large sample size study suggest that PSB is not a rare condition in people living in Turkey. In accordance with previous studies from other countries being female and being younger seemed to increase the risk of PSB. Symptoms of psychiatric distress, negative and positive affect were positively correlated with PSB in our sample as has been shown in previous studies on problematic shopping addiction.

Our findings suggest that further research is needed to understand different motives that underlie the problematic shopping behavior in the young and female population in comparison to older and male populations. Any preventive measure for PSB needs to target relatively young and female populations primarily. These preventive programs or any interventions for people with PSB needs to address regulation difficulties and development of healthy strategies to cope with psychiatric distress. Regarding our findings on the attachment style and SA, it can be said that these people might benefit from Interpersonal psychotherapy[69].

## Supplementary Information

**Additional file 1**. Shopping addiction risk questionnaire (SHARQ).

## Data Availability

The datasets used and/or analysed during the current study available from the corresponding author on reasonable request.
