# Supplementary material for: The prevalence and psychological relation of problem shopping: data from a large-scale sample from Turkey
Source: BMC Psychol. 2022 Jan 3;10:1. doi: 10.1186/s40359-021-00711-6 (PMC8722422; doi:10.1186/s40359-021-00711-6)
Supplement: Supplementary file 1 — Additional file 1. Shopping addiction risk questionnaire (SHARQ). [file 40359_2021_711_MOESM1_ESM.docx]

**APPENDIX**

**Please rate below items considering your shopping behavior.**

| 1. 1. This behavior has become the center of my life. If I can't, I think when I can. Sometimes there is such a strong urge that I find a way to engage in this behavior and I can't stop it (Salience) |
| --- |
| 1. Sometimes when I am bored, sometimes when I am happy, I think and do this behavior. Often times, when my problems increase, my desire to engage in behavior increases (Mood modification) |
| 1. Even if I do the behavior at the same rate, sometimes it is not enough for me, I need to be busier. I need to increase the amount and time I engage in the behavior or I won't relax. (Tolerance) |
| 1. I feel signs of tension, psychologically or physically, when I am in a place where it is impossible for me to do this Behavior, or when I try to stop myself or when someone sees my problem and tries to intervene (Withdrawal) |
| 1. Doing this behavior causes me to have problems around me, my social life is affected badly, it can hinder my work and I am often criticized for this Conflict) |
| 1. If I stop doing this behavior, it may trigger again and again and it will be as if I never stopped (Relapse) |
